# Exon and junction microarrays detect widespread mouse strain- and sex-bias expression differences

**DOI:** 10.1186/1471-2164-9-273

**Published:** 2008-06-04

**Authors:** Wan-Lin Su, Barmak Modrek, Debraj GuhaThakurta, Stephen Edwards, Jyoti K Shah, Amit V Kulkarni, Archie Russell, Eric E Schadt, Jason M Johnson, John C Castle

**Affiliations:** 1 Molecular and Cellular Biology Program, University of Washington, Seattle, WA 98195, USA; 2 Rosetta Inpharmatics LLC, a wholly owned subsidiary of Merck & Co., Inc., 401 Terry Ave N, Seattle, WA 98109, USA

## Abstract

**Background:**

Studies have shown that genetic and sex differences strongly influence gene expression in mice. Given the diversity and complexity of transcripts produced by alternative splicing, we sought to use microarrays to establish the extent of variation found in mouse strains and genders. Here, we surveyed the effect of strain and sex on liver gene and exon expression using male and female mice from three different inbred strains.

**Results:**

71 liver RNA samples from three mouse strains – DBA/2J, C57BL/6J and C3H/HeJ – were profiled using a custom-designed microarray monitoring exon and exon-junction expression of 1,020 genes representing 9,406 exons. Gene expression was calculated via two different methods, using the 3'-most exon probe ("3' gene expression profiling") and using all probes associated with the gene ("whole-transcript gene expression profiling"), while exon expression was determined using exon probes and flanking junction probes that spanned across the neighboring exons ("exon expression profiling"). Widespread strain and sex influences were detected using a two-way Analysis of Variance (ANOVA) regardless of the profiling method used. However, over 90% of the genes identified in 3' gene expression profiling or whole transcript profiling were identified in exon profiling, along with 75% and 38% more genes, respectively, showing evidence of differential isoform expression. Overall, 55% and 32% of genes, respectively, exhibited strain- and sex-bias differential gene or exon expression.

**Conclusion:**

Exon expression profiling identifies significantly more variation than both 3' gene expression profiling and whole-transcript gene expression profiling. A large percentage of genes that are not differentially expressed at the gene level demonstrate exon expression variation suggesting an influence of strain and sex on alternative splicing and a need to profile expression changes at sub-gene resolution.

## Background

Variation in mammalian mRNA expression is significantly affected by both strain and sex. In mice, widespread sex differences have been observed in adipose, kidney, liver, muscle, and brain tissue [1-3]. Given the heterogeneity of brain tissues, extensive studies have also been performed in various parts of the brain, highlighting the extent of sexual dimorphism in the hypothalamus, pituitary gland, and the cortex [1]. In mice, the estimate of inter-strain variation in various regions of the brain is as high as 30% [4].

While alternative splicing has been acknowledged to play an important role in genetic diversity, few large-scale studies have investigated the effects of strain, sex and tissue on exon expression or other alternative splicing mechanisms. Such studies have, for example, identified splicing events involved in cancer and tissue definition [5,6]. In *Drosophila*, where sex-specific splicing has long been shown to be involved in sex determination, 22% of alternatively spliced genes demonstrated sexual dimorphism [7]. Further studies on alternative splicing have demonstrated that some single nucleotide polymorphisms (SNPs) are responsible for variations in the ratios of alternative spliced transcripts [8-11]. Given that at least 8 million SNPs exist in the mouse population [12], we decided to investigate the effects of differing genetic backgrounds and sex on alternative splicing patterns in a mammalian system.

In this study we present a broad survey of the role of naturally occurring genetic variations and sex differences upon gene expression and splicing in liver, a key tissue regulating many disease conditions such as metabolic disorders and cardiovascular disease. We profiled the expression of 9406 exons representing 1020 genes in 71 female and male mouse livers from strains DBA/2J, C57BL/6J, and C3H/HeJ. Genes were selected for involvement in obesity, diabetes, cardiovascular diseases, and bone traits [13-19] and strains were chosen based on their distinct genealogy [20]. We selected a single representative transcript for each gene (see methods for details) and designed a custom microarray with exon and exon-junction probes for each exon, enabling us to investigate expression changes at both the gene and exon levels. In addition, we examined the differences between profiling gene expression using a single probe at the 3' end vs. using multiple probes spaced along the gene.

Our results indicate a degree of concurrence between 3' gene and whole-transcript gene expression profiling: over 64% of the genes identified as significantly differentially expressed using 3' gene expression profiling methods are also identified through whole-transcript gene expression profiling. Moreover, whole-transcript gene expression profiling identified 13% to 20% more differential expression than 3' gene expression profiling. Exon expression profiling, however, identified at least 38% or more genes with at least one differentially expressed exon. 55% and 32% of genes showed differential exon expression by strain and sex, respectively. Finally, by examining gene expression at the sub-gene resolution, we found 205 genes to exhibit differences in exon expression for both strain and sex.

## Results

We profiled 9406 exons representing 1020 genes using male and female liver samples from three strains, analyzed the results at three distinct levels: 3' gene expression, whole-transcript gene expression, and exon expression. Microarray data were deposited at GEO under GSE10736.

### Sex differences are larger than strain differences

**Figure 1:**
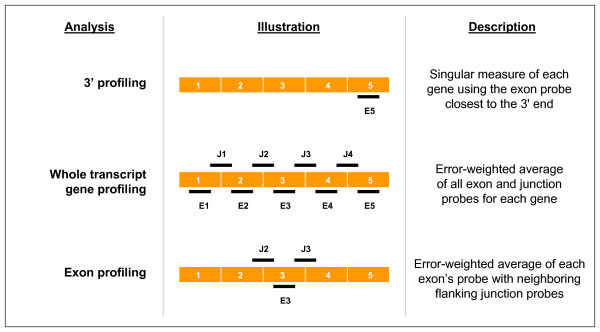
**Cartoon illustration**. Description and cartoon illustration of the datasets modeled. See Materials and Methods for a detailed explanation of each set.

**Figure 2:**
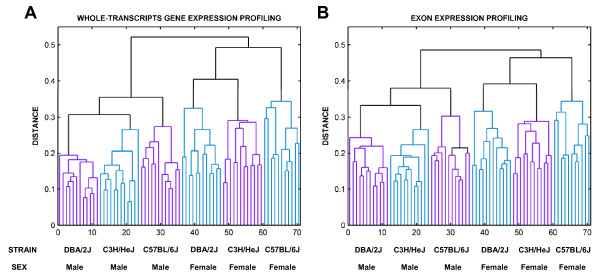
**Hierarchical clustering of gene and exon expression**. A) Mouse clustering (agglomerative clustering) based on gene expression profiling. B) Mouse clustering (agglomerative clustering) based on exon expression profiling.

With each dataset described in Figure 1, we performed hierarchical clustering using Pearson correlation as a measure of similarity. Similar dendrograms are observed for all three datasets indicating that the relative magnitude of variation within groups and between groups is similar within each dataset (see Figure 2 and Additional file 1). Among the strains, sex differences were larger than strain differences (Figure 2). Within each sex, the samples clustered by strains with DBA/2J samples being closer to C3H/HeJ samples than to C57BL/6J. We then computed an averaged gene expression profile for each strain-sex combination using all probes and computed the correlation between groups. On average, gene expression within males are more closely correlated than in females (r = 0.83 in males vs. r = 0.67 in females). The correlation coefficient between males and females within the same strain ranges from r = 0.56 to 0.64 (see Additional file 2).

**Figure 3:**
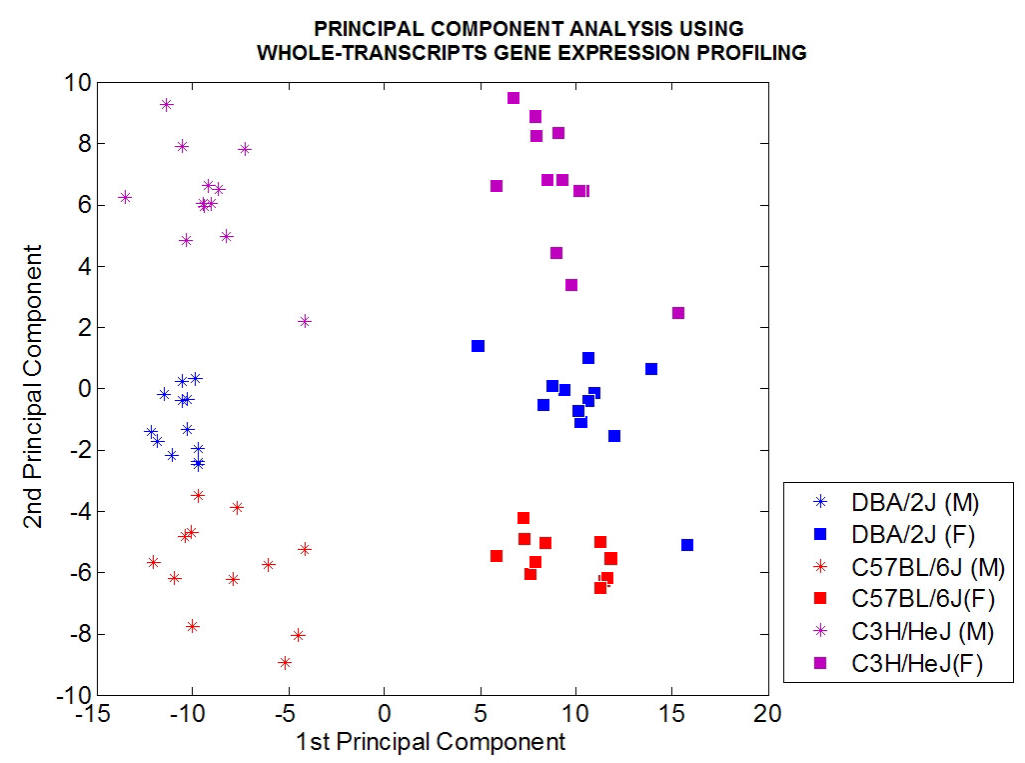
**Principal component analysis of whole-transcript gene expression**. Principal component analysis using the whole-transcript gene expression values that are differentially expressed between sexes.

Similarly, using the differentially expressed genes between sexes as markers, principal component analysis is able to completely separate tissues into six distinct groups representing each strain-sex population (Figure 3). Similar results are obtained using differentially expressed exons. Using the mean log_10 _expression values from exon expression profiling, the sum of the first two principal components account for 51% of the total variance observed for sex-biased genes.

### SNPs affect probe intensities

We were able to map 97% of all the probes to the Mouse Genome NCBI Build 36. Using recent genotype data [12], we identified over 1.5 million SNPs present within C57BL/6J, DBA/2J, and C3H/HeJ mice. Only 3% of all probes overlap a SNP from one of these strains. To test the effects of the presence of SNPs in probes, we performed a two-way factorial ANOVA designed using strain and sex as factors. Of the 3421 probes that were differentially expressed, 194 (6%) had at least one SNP within the probe region. Conversely, only 339 out of 14510 non-differentially expressed probes contained at least one SNP. Hence the enrichment for SNPs within differentially expressed probes is very significant at a Fisher's exact test p-value of 1.3 × 10^-21 ^and an odds ratio of 2.51. Furthermore, junction probes overlapping SNPs have a higher odds ratio (2.83 vs. 2.31) of being differentially expressed compared to exon probes overlapping SNPs (see Additional file 3). Because a change in probe intensity could reflect either a change in alternative splicing or a change in the binding affinity, due to the SNP, we decided to discard probes overlapping SNPs from these strains.

### Exon profiling identifies more differences

**Figure 4:**
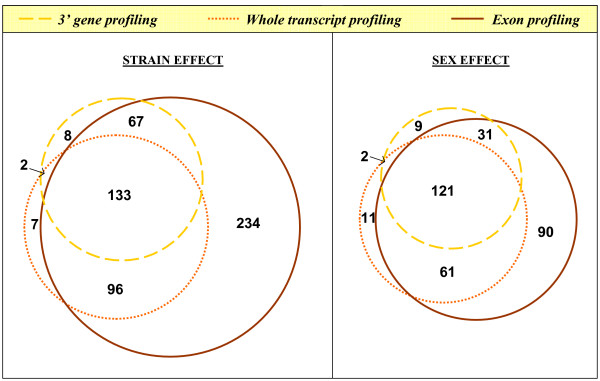
**Comparison of 3' gene expression profiling, whole-transcript gene expression profiling and exon expression profiling**. Venn diagram illustrating the overlap between genes identified using 3' gene expression profiling, whole-transcript gene expression profiling and genes associated with an exon identified using exon expression profiling for strain- and sex-bias expression.

We tested genes and exons for differential expression using a two-way factorial ANOVA design using strain and sex as factors (Table 1). Using 3' gene expression profiling techniques, 22% and 17% of the genes showed significant strain and sex effects. At the same false positive rate (Bonferroni corrected p-value < 0.01), whole-transcript gene expression profiling identified 25% and 20% of the genes to be differentially expressed between strain and sex, respectively. Examining the overlap between methods, 64% and 73% of strain- and sex-biased genes identified via 3' gene profiling were detected by whole-transcript profiling and whole-transcript profiling identified 13% and 20% more genes than 3' gene profiling (Figure 4).

**Table 1:** Number and percentage of differentially expressed genes in each dataset (Bonferroni corrected p-value < 0.01).

			**Exon profiling**	
			
**Effects**	**3' profiling (n = 941)**	**Whole-transcript gene profiling (n = 941)**	**Exons (n = 9055)**	**Associated Genes (n = 941)**
Strain	210 (22%)	238 (25%)	1751 (19%)	520 (55%)
Sex	163 (17%)	195 (20%)	1261 (14%)	303 (32%)

*Note that the right column under exon expression profiling is the number of unique genes represented by the set of differentially expressed exons for each effect. The numbers in parentheses in the first row represents the total number of genes or exons profiled.

When analyzed at the exon level, we found 19% and 14% of exons are differentially expressed across strain and sex, respectively, using the same Bonferroni-corrected p-value threshold of 0.01. In terms of genes associated with these differentially expressed exons, 55% and 32% of genes have at least one differentially expressed exon across strain and sex, respectively. Overall, 3014 exons (33%), representing 823 genes (87%), showed significant strain- or sex-specific biases in expression. Surprisingly, when we tested each gene for sex-strain interaction effects, we found only 1% to 2% of genes (depending on the datasets used) have significant interaction effects at a Bonferroni-corrected p-value threshold of 0.01, close to the level expected by chance.

We then analyzed the overlap between genes identified as differentially expressed using gene profiling analysis and exon profiling analysis. 95% and 93% of strain-bias and sex-bias genes identified by 3' gene profiling were identified in exon profiling. Similarly, 96% and 93% of strain-bias and sex-bias genes identified via whole-transcript gene profiling were detected via exon profiling. However, 234 and 90 genes containing an exon showing strain-bias and sex-bias effects, respectively, from exon profiling were not detected as differentially expressed by either gene expression profiling method. Thus, 75% and 38% of the genes with differences were identified only by exon profiling.

### Variation across strain and sex

**Figure 5:**
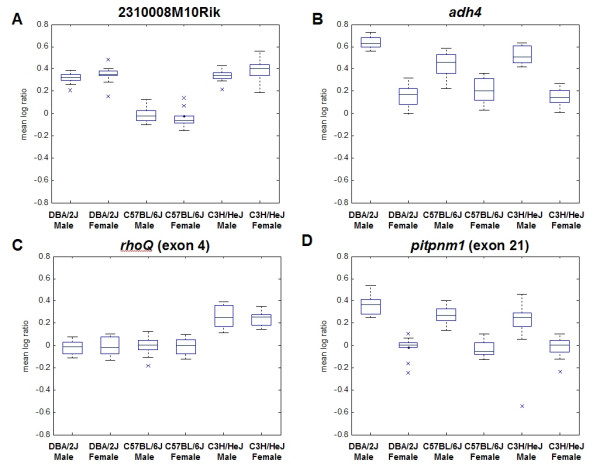
**Examples of differentially expressed genes and splice forms**. Box plots of the mean log ratio obtained from multiple probes in each strain-sex group for a single gene. The solid horizontal line across the length of the graph represents the average gene expression. The two dashed horizontal lines mark plus and minus two standard deviations from the average gene expression, respectively. For the boxes, from bottom to top, the solid horizontal lines represent the minimum mean log_10 _expression ratios excluding outliers, the lower quartile, the median, the upper quartile and the maximum mean log_10 _expression ratios excluding outliers. The dashed vertical lines represent the range of mean log_10 _expression ratios excluding outliers. Outliers are defined as any data-points having values extending beyond 1.5 times the interquartile range from either ends of the box. A) Box plots of 2310008M10Rik showing strain effect (p-value < 1 × 10^-16^); B) Box plot of *adh4 *showing sex effect (p-value < 1 × 10^-16^). C) Boxplots of *rhoQ*, exon 4 showing strain effect (p-value < 1 × 10^-16^); D) Boxplots of *pitpnm1*, exon 21 showing sex effect (p-value < 1 × 10^-16^)

Strain- and sex-bias genes detected via exon expression profiling comprise genes differentially expressed at the gene level and differential alternative splicing. For example, 2310008M10Rik (aDC2-like protein, NM_025509) and *adh4 *(NM_011996, alcohol dehydrogenase 4 (class II) pi polypeptide) both show differential expression at the gene level. In the case of 2310008M10Rik, each exon demonstrates strain-biased expression (Figure 5A). 2310008M10Rik is down-regulated at the gene level in C57BL/6J relative to DBA/2J and C3H/HeJ and this phenomenon is consistent across both males and females. Similarly, each exon in *adh4 *shows sex-biased expression and is up-regulated in males across all strains (Figure 5B). In both cases (2310008M10Rik and *adh4*), all exons were identified as differentially expressed between strains and sexes respectively in the exon expression profiling dataset.

234 and 90 genes showed strain- and sex-bias effects, respectively, for differences in exon expression in genes not identified as differentially expressed using 3' gene expression or whole-transcript gene expression profiling. For example, exon 4 in *rhoQ*, which encodes ras homolog gene family, member Q protein, showed significantly lower mean log_10 _ratios in DBA/2J and C57BL/6J while demonstrating elevated expressions in C3H/HeJ relative to the pool of control samples suggesting that different ratios of alternatively spliced isoforms are present among the three strains (Figure 5C). Similarly, exon 21 in *pitpnm1 *(phosphatidylinositol membrane-associated 1) showed higher expression levels in males relative to females in all three strains (Figure 5D) strongly suggesting that at least two different forms or isoform ratios of *pitpnm1 *are expressed between males and females.

We found many expression changes associated with sex and thus investigated if the X chromosome was enriched for differentially expressed genes or splicing events. Using a Fisher's exact test, we found no enrichment for differentially expressed genes or differential splicing on the X chromosome (p-value > 0.9, see Additional file 4).

**Figure 6:**
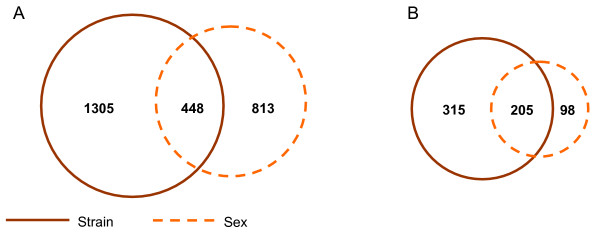
**Genes showing both strain- and sex-biased effects**. Venn diagrams showing A) the overlap between exons showing strain effects and those with sex effects and B) the overlap between genes with differentially expressed exons showing strain effects and those with sex effects. For the diagram shown in (B), we restrict the analysis to genes that utilizes different exons for strain- and sex-bias expression (see text).

As strain and sex are major factors influencing gene expression, we next asked how many genes showed both strain- and sex-bias effects via differential exon expression of the same or different exons within a gene. 17% of differentially expressed exons (448 exons) exhibit both strain and sex biases (Figure 6A) and were found in 137 genes. We then examined the number of genes with multiple exons showing differential expression, where an exon demonstrating strain-bias expression is distinct from those showing sex-bias expression. 22% of differentially expressed genes (205 genes) demonstrated both strain- and sex-biases at the exon level (Figure 6B).

## Discussion

Messenger RNA gene expression variation can be divided into two broad categories: differences in the overall mRNA level (due to transcriptional changes or mRNA stability) and alterations in the ratios of alternative transcripts. Variations in gene expression can be due to a number of different factors, including genetic variation, epigenetic variation, environment variation (which could include, for example, the hormonal differences between males and females) and interactions between these factors. In this paper, we first quantified the contributions to differential expression from two sources of variation – strain and sex – and secondly, provided evidence suggesting that variation in transcript structure contributes significantly to mRNA expression variation.

When we clustered the dataset using hierarchical methods, we found that the mice of the same sex, but different strains, were more similar in terms of gene expression than mice in the same strain but of different sex. Furthermore, after this initial subdivision into males and females, the phylogenetic tree obtained matches that shown in previous SNP-based genealogy studies[19,20]. Thus, we conclude that (a) the natural variation of gene and exon expression is smaller between mouse populations of similar sex than that of the same strains but differing sexes, and (b) to the extent we have examined, gene and exon expression captures the differences identified by genealogy. Based on genealogy of mouse strains, we believe more differences in gene expression and splicing could have been observed if the strain selection had been even more diverse, such as the inclusion of strains 129S1/Svlm, and SWR/J [19,21].

If we compare our estimates of strain-bias genes from 3' gene expression profiling to those found in the literature, our estimates of 23% fall within the range that has been documented by others. For example, Nadler et al. (2006) found 57% of genes exhibited strain-biases at the level of gene expression whereas Pavlidis and Noble (2001) and Sandberg et al. (2000) found only 1% to 2% of genes show inter-strain variation [4,22,23]. As pointed out by Nadler et al. (2006), the higher estimates are likely due to the inclusion of a larger more diverse set of strains, i.e., 10 strains in Nadler et al. (2006) study vs. 2 strains in Sandberg et al. (2000) [4,23]. In addition, differences in the tissues examined are also likely to significantly influence variance estimates, since the studies mentioned above used brain tissue while our study was performed using liver. In terms of sex estimates, we find that our estimate, at 17%, is markedly smaller than Yang et al. [3]. This difference likely reflects the dramatic differences in power between the two studies to detect expression differences, given only 11 or 12 animals were profiled from each sex for each strain in or study, versus the more than 150 individuals per sex profiled in the study by Yang et al.

We detected significant variation in exon expression with regards to the genetic background and sex. While the probes used for exon expression profiling may be more susceptible to cross-hybridization and higher background levels given the smaller target regions, the use of cDNA amplification products partially mitigates this effect [24]. Furthermore, by averaging multiple exon and junction probes, we increased the reliability of each measurement and reduce the impact of individual SNPs. However, averaging exon and junction probes makes it more difficult to distinguish different types of splicing events. Nevertheless, through the use of exon expression profiling technologies, we were able to detect 234 and 90 genes with strain- and sex-bias effects, respectively, that were not detected in the 3' gene expression or whole-transcript gene expression profiling analysis. These numbers suggest that many alternative splicing events are differentially expressed but go undetected by current gene expression profiling technologies.

Splicing differences between groups can be attributed to genetic or epigenetic variation. For example, variations in *cis*-acting regulatory elements, such as SNPs within promoter sequences, splicing enhancers or splicing silencers can alter transcriptional initiation rates and splicing patterns. Structural variations in *trans*-acting splice regulatory proteins may affect global splicing patterns and nucleotide variation in mRNA transcripts can influence translational efficiency (as shown with apolipoprotein A-II in mice [25]) and/or mRNA decay rates. Expression and splicing differences observed between different sexes, however, showcase the amount of underlying biological mechanisms that have yet to be elucidated. With the exception of the sex chromosomes, the genome is essentially identical between the males and females of an inbred mice strain, hence the possible mechanisms that give rise to gene expression and/or splicing variation include trans-acting factors on the sex chromosomes (such as SRY or the Sox proteins), epigenetic variations, and/or hormonal differences.

Oligonucleotide probes overlapping SNPs are biased towards differential expression, leading to overestimation of differential expression. A study using probes from the Affymetrix platform recently demonstrated the susceptibility of single probes to SNPs and highlights the impact of natural variation on hybridization based methods [26]. We found similar findings in longer 36 nt and 60 nt probes. For example, we found that 36 nt junction probes overlapping a SNP show higher sensitivity towards differential expression, possibly due to alternative splicing brought about by SNPs within splice sites or due to differences in probe binding affinities due to the SNP.

We have confirmed that gene expression is significantly affected by strain and sex and provided evidence suggesting that this effect extends to alternative splicing which, to our knowledge, had not been shown in mammals. Given that variations in alternative splicing patterns lead to a wide variety of downstream biological effects, our results provides further justification for investigations on alternative splicing variations in genetically segregating populations.

## Conclusion

A large degree of strain- and sex-bias variation is observed in mouse liver tissue. Differences are observed both at the overall mRNA levels and in the expression of individual exons. We estimate 55% and 32% of genes demonstrate differential expression between strains and sex, respectively, at the level of gene or exon expression. Exon expression profiling captures the majority of genes identified by 3' gene expression profiling (93%), and identifies many more genes containing differential exon expression – changes invisible to 3' gene expression profiling. In these samples, by profiling only the 3' end of each gene and not exons, more than half the biological information present in the mRNA variation is lost.

To our knowledge this is the first study reporting a broad survey of the strain and sex effects upon individual exon expression. Genetic variation of gene expression has been used by several groups, including ours, in studying the genetic causes of complex disease and the identification of causative genes for such diseases [14,15,19,27,28]. Recently, Kwan et al. (2007) identified significant association between alternative splicing patterns and cis-regulatory regions in humans demonstrating the heritability of alternative splicing events [29]. These studies along with our finding that changes in exon expression is widespread between mice of difference strains demonstrate the importance of monitoring variations in splice forms and that splicing is affected broadly by natural genetic variations.

## Methods

### Mice background and RNA collection

C57BL/6J, C3H/HeJ, and DBA/2J mice were reared at JAX in Bar Harbor, Maine and shipped to JAX in Sacramento, California at 7 weeks of age. A total of 12 mice for each strain and each sex were placed on a standard chow diet for 12 weeks. Mice were kept in similar environmental conditions to minimize environmental effects. Female mice were not synchronized with respect to estrogen cycle. At 20 weeks of age, all mice were euthanized and liver tissue was collected at necropsy and flash frozen in liquid nitrogen. One liver sample failed quality control, resulting in a total of 71 mouse livers profiled.

### Array design

From previous studies in our lab and information found in the published studies, we selected 1,312 mouse genes. For each gene, a representative transcript was selected, with priority to longer RefSeq NM transcripts, followed by Genbank mRNAs, and lastly dbEST ESTs. Each transcript was aligned to the Celera mouse genome sequenced to define the exon structure [30]. Probes were selected as in Johnson et al. (2003) [31], where 36 nt junction probes were placed across exon-exon junctions with 18 nt in each exon, and synthesized on 26 nt stilts (60 nt total) and an optimal 60 nt probe was selected for each exon. Custom-designed exon and junction microarray, containing 25760 probes representing 1312 genes, were transmitted to and printed by Agilent Technologies (California).

### Preparation of labeled cDNA, array hybridization, experimental design, and image processing

Hybridization material was generated through a random-priming amplification of poly [A]+ purified RNA using primers with a random sequence at the 3' end and a fixed motif at the 5' end that was optimized to generate strand-specific cDNA copies of full-length mRNA transcripts [32]. Since the region used for exon and junction probe selection is constrained to a smaller region, more probes contain sequence with suboptimal characteristics (e.g. high GC content or higher homology to other genes). The hybridization of cDNA, rather than cRNA as commonly done, partially mitigates this issue due to higher specificity and lower background levels [24].

Hybridization conditions were as previously described [33]. All 71 samples were hybridized in a two-channel experiment, where one channel was a common reference, generated by pooling all 71 samples in equal mass. Array hybridizations were done in duplicate with fluor reversal to systematically correct for Cy3/Cy5 dye bias. Array images were processed as described to obtain background noise, single channel intensity and associated measurement error estimates [34]. Expression changes between samples and pool were quantified as mean log_10 _(expression ratio), and associated error.

### Gene and exon expression

The expression dataset was first filtered to exclude probes with saturated intensities or those below background levels. Based on previous experiments with the ink-jet microarray platform and Agilent scanner, we flagged probe intensities that fell outside of the linear range, either near saturation or background levels. The filtered dataset contained expression ratios for 1020 genes represented by 9406 exons. We calculated gene expression in two ways; using a single 3' probe to mimic commonly used 3' based microarray profiling and using the mean of all probes measurements associated with the gene. Standard microarray experiment utilizes an oligo-dT based amplification protocol that amplifies only the region immediately 5' of the poly adenylation site. Probes on standard arrays are thus situated near the genes' 3' terminus. As a surrogate for standard 3' gene expression profiling, we selected the exon probe located closest to the 3' end on our custom-design arrays and extracted the mean log_10 _ratio to the reference pool; forming a "3' gene expression profiling" dataset. We also calculated a "whole-transcript" gene expression dataset using all exon and junction probes along the entire length of the gene. The mean log10 ratios for all probes associated with a given gene were then averaged forming a single measurement for the transcript. For exon expression profiling, we again used the average of the mean log_10 _ratios of each exon probe and the two flanking junction probes. In the case of the first or last exon, only the exon probe and a single flanking junction probe was used (Figure 1).

Subsequent analysis performed required the removal of probes with SNPs within the probe body. Hence, for the 3' gene expression profiling dataset, if the exon probe closest to the 3' end overlapped SNPs, we discarded the gene. For whole transcript gene expression profiling, we computed the average measurement of all probes with no SNPs and used only exons and flanking junction probes containing no SNPs for exon expression profiling.

### Statistical analysis

Genes and exons were clustered in 1-dimension using agglomerative methods with Pearson correlation being used as a measure of similarity. Correlation values between groups were computed by first averaging the mean log10 expression values from all individuals in each group, and then calculating the Pearson correlation using all complete pairwise values for each gene between all possible group pairs. In terms of differential expression, each dataset was analyzed using the following two-way analysis of variance (ANOVA) model:

*Y*_*ijk *_= *μ*_*k *_+ *S*_*ik *_+ *G*_*jk *_+ *ε*_*ijk*_

where *Y*_*ijk *_is the value for strain *i*, sex *j *and gene *k*; *μ *is the overall mean; *S*_*i*_, and *G*_*j *_represent the strain effects for strain *i *and sex effects for sex *j *respectively; and *ε *_*ijk *_is the error term. The results were then adjusted for multiple hypothesis testing using Bonferroni correction (see Additional files 5, 6, 7). For each main effect, genes and exons with a Bonferroni corrected p-values of less than 0.01 were identified as differentially expressed. Initially, we specified a sex-strain interaction term. However, only 1% to 2% of genes have significant sex-strain interaction effects at a Bonferroni-corrected p-value threshold of 0.01. Since this is either slightly higher or exactly what we would expect by chance alone, we discarded the term in favor of a more precise model.

### SNP analysis

A set of 1,533,914 SNPs were obtained from the mouse resequencing project by Perlegen-US National Institute of Environmental Health Sciences [12]. These SNPs represented the complete set of polymorphisms between C57BL/6J, DBA/2J and C3H/HeJ where at least one pair was polymorphic for each SNP. No missing data was permitted in the SNP set. Probes with at least one SNP were then identified. Using the same ANOVA model described above, we identified probes that were differentially expressed between strains, again using a Bonferroni-corrected p-value threshold of 0.01. Fisher's exact test was then used to determine if differentially expressed probes were significantly enriched for probes containing SNPs. This analysis was repeated for our 3' gene expression profiling dataset. A similar analysis was performed for the whole-transcript gene expression profiling dataset using all probes associated with the transcript. For exon profiling, because a junction probe often overlaps with both a differentially expressed exon and a non-differentially expressed exon, we associated each exon with the number of SNPs within the exon probe and its flanking junction probe. Results of the above analysis are summarized in Additional files 3, 4 and 8.

## Authors' contributions

W–LS and JCC wrote the manuscript with edits from all co-authors. W–LS designed and performed the data analysis with feedback from JCC and EES. BM designed the experiment. DGT, SE, and JCC designed the microarray patterns. JKS performed the overlap with EST splice variants. JKS and AR mapped probes onto the current build. AVK deposited the data in GEO. EES and JMJ conceived the project. All authors have read and approved the manuscript

## Supplementary Material

Additional file 1 Hierarchical clustering of 3' expression profiling dataset. Mouse clustering (agglomerative clustering) based on 3' gene expression profiling. Click here for file

Additional file 2 Correlation between strain-sex groups. Pearson correlation using the average whole transcript profiling log10 ratios from each group. Click here for file

Additional file 3 SNP Analysis. Fisher's exact test for enrichment of probes containing SNPs within differentially expressed probes. Click here for file

Additional file 4 Sex chromosome Analysis. Fisher's exact test for enrichment of differentially expressed probes on the X chromosome. Click here for file

Additional file 5 ANOVA p-values for 3' gene expression profiling dataset. List of genes, raw p-values and Bonferonni-corrected p-values using the 3' gene expression profiling dataset. Click here for file

Additional file 6 ANOVA p-values for whole transcript gene expression profiling dataset. List of genes, raw p-values and Bonferroni-corrected p-values using the whole transcript gene expression profiling dataset. Click here for file

Additional file 7 ANOVA p-values for exon expression profiling dataset. List of genes and associated exons, raw p-values and Bonferroni-corrected p-values using the exon expression profiling dataset. Click here for file

Additional file 8 Analysis of exon probes near splice sites. Fisher's exact test for enrichment of differentially expressed probes within exon probes that overlap with junction probes. Click here for file
