# Supplementary material for: Exon and junction microarrays detect widespread mouse strain- and sex-bias expression differences
Source: BMC Genomics. 2008 Jun 4;9:273. doi: 10.1186/1471-2164-9-273 (PMC2432077; doi:10.1186/1471-2164-9-273)
Supplement: Additional file 1 — Hierarchical clustering of 3' expression profiling dataset. Mouse clustering (agglomerative clustering) based on 3' gene expression profiling. [file 1471-2164-9-273-S1.pdf]

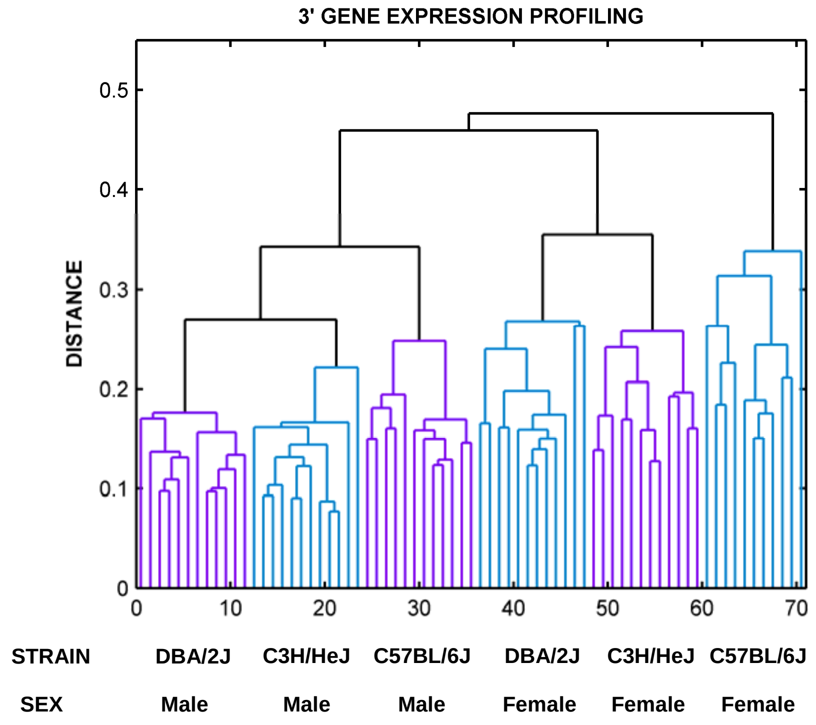
3' GENE EXPRESSION PROFILING
DISTANCE
0.5
0.4
0.3
0.2
0.1
0
0
10
20
30
40
50
60
70
STRAIN
DBA/2J
C3H/HeJ
C57BL/6J
DBA/2J
C3H/HeJ
C57BL/6J
SEX
Male
Male
Male
Female
Female
Female
